# Determinants of Allergic Rhinitis in Young Children with Asthma

**DOI:** 10.1371/journal.pone.0097236

**Published:** 2014-05-15

**Authors:** Lise Moussu, Philippe Saint-Pierre, Virginie Panayotopoulos, Rémy Couderc, Flore Amat1, Jocelyne Just

**Affiliations:** 1 Allergology department, Centre de l′Asthme et des Allergies, Hôpital d'Enfants Armand-Trousseau (Assistance Publique Hôpitaux de Paris) - Université Pierre et Marie Curie, Paris, France; 2 Laboratoire de Statistiques Théoriques et Appliquées, Université Pierre et Marie Curie, Paris, France; 3 Service de Biochimie et Biologie Moléculaire, Hôpital d'Enfants Armand-Trousseau (Assistance Publique Hôpitaux de Paris), Paris, France; Beijing Institiute of Otolaryngology, China

## Abstract

**Background:**

In the preschool period, allergic rhinitis (AR) is infrequent and thus under-diagnosed. However, recent works have highlighted the occurrence of AR in toddlers although the causes of AR in this young population remain unknown. The objective of this study was to identify determinants of AR in young children with asthma.

**Methods:**

We carried out a case-control study of 227 children with active asthma and enrolled in the Trousseau Asthma Program. AR and other allergic diseases (asthma, food allergy and eczema) were diagnosed by medical doctors using standardized questionnaires. Parental history of AR and asthma, biological markers of atopy (total IgE, blood eosinophilia, allergic sensitization towards food and aeroallergens) and environmental parameters were also collected.

**Results:**

Forty one of the children (18.1%) had AR. By univariate logistic regression analysis, AR was mainly associated with peanut sensitization (OR = 6.75; p = 0.002); food allergy (OR = 4.31; p = 0.026); mold exposure (OR = 3.81 p<0.01) and parental history of AR (OR = 1.42; p = 0.046). Due to the strong link between food allergy and peanut sensitization three models of multivariate logistic regression were performed and confirmed that AR is associated with peanut sensitization but also food allergy and mold exposure. A random forest analysis was also performed to explain AR. The results reinforced the logistic analysis that peanut sensitization and mold exposure were the principal determinants of AR.

**Conclusions & Clinical Relevance:**

These results stress the importance of investigating AR in young children with asthma to potentially diagnose a particularly severe allergic asthmatic phenotype. Moreover, these data evoke the hypothesis that peanut could be an aeroallergen.

## Introduction

Allergic rhinitis (AR) is an atopic manifestation frequently associated with asthma. The prevalence of AR in children has been rising in many countries and is associated with a western lifestyle [1]. The Score for AR (SFAR) [2], which has been validated in the pediatric population after adding the idea of nasal itching [3], is a valuable diagnostic tool. The AR and its Impact on Asthma (ARIA)[4] classification further helps assess the severity of this pathology, as it takes into account the chronology of the symptoms and the discomfort they cause. In addition to causing other ear, nose and throat disorders [5] and harmful effects on quality of life [6], AR has been identified as a risk factor for asthma onset [7], [8], severity and poor control [9]. The occurrence of AR in pre-school children is now widely accepted [10]–[12], but diagnosis has long been overlooked or questioned in early infancy. This is mainly explained by its similarity with infectious symptoms which are frequent in toddlers.

The « Pollution and Asthma Risk: an Infant Study » (PARIS) birth cohort, gave an estimated general prevalence of AR of around 9.1% in 18-month-old infants [13], but other studies give estimates ranging from 3 to 29%, depending on how stringent the applied criteria were [14], [15]. AR and asthma are two components of the chronic allergic respiratory syndrome, as they involve the same allergic immunological processes, fostering the concept of unity of the respiratory tract [16]. Nevertheless, because allergen sensitization of asthmatic infants seldom occurs, except for infants having an allergic phenotype [17], the mode of its association with AR is still unknown at this age of life.

Our study set out to identify determinants associated with AR in a case-control study performed in a cohort of young children with asthma.

## Materials and Methods

### Ethics

Data were collected by standardized questionnaires and the medical examination was conducted by a physician after the parents gave their written informed consent. The Ile de France V ethics committee approved the protocol and this consent procedure.

### Population

This case-control study gathered data from January to November 2011 and enrolled children who were part of the TAP (Trousseau Asthma Program). All the children had been referred to the center for recurrent wheezing by a primary care physician and joined the study consecutively.

Inclusion criteria for the study were: age younger than 30 months; persistent asthma (defined as a history of recurrent wheeze, more than three episodes of reversible bronchial obstruction documented within the previous 6 months) [18]; absence of other chronic obstructive pulmonary diseases (congenital or acquired), or exacerbation or acute respiratory illness within the 6 weeks preceding the explorations.

### Health outcomes

General data were collected on the children's gender and age.

AR was diagnosed by a medical doctor supported by the SFAR criteria as defined by a score of 7 or more [3]. Based on these data, infants were allocated into two groups – one comprising children diagnosed with AR (the AR group) and the other comprising those without (the control group) – in a randomized manner and adjusted for age and gender.

Personal history of atopy (eczema) was mostly assessed by questions from the International Study of Asthma and Allergies in Childhood (ISAAC) [19]. Food allergy was defined by clinical symptoms of allergy after consumption of a food allergen and a positivity of specific IgE for the same allergen.

Moderate to severe asthma was determined according to the National Asthma Education and Prevention Program [20].

Finally, information about parental history (paternal and/or maternal) of AR and asthma were also gathered.

### Environmental factors

Environmental factors were assessed by the questions applied in the PARIS birth cohort. We collected data about exclusive maternal breast-feeding beyond 3 months, exposure to passive smoking (defined by smokers at home smoking more than five cigarettes a day) and overcrowding (less than 9 m^2^ for each person living at home). Potential sources of biologic allergens were also evaluated: mold exposure (visible mold and moldy smell) and presence of visible cockroaches at home.

### Biological markers

Biological markers of atopy were measured in peripheral blood. These included the multiple allergenic screening test Phadiatop infant and blood levels of specific IgE toward inhaled and food allergens (cow milk, egg, peanut, house dust mite, grass pollen, birch pollen, cat, dog and alternaria) for children testing positive. Children with two or more sensitizations to food and/or aeroallergens were considered to have polysensitization. Aeroallergen sensitization was defined as at least one sensitization to an inhaled allergen. Other inflammatory biological markers, such as total eosinophilia count and serum level of total IgE, were measured too. Thresholds were used to define increased levels: increased blood eosinophilia was defined as a concentration ≥470 eosinophils/mm^3^, (cell count by automated Sysmex; France), increased total IgE as a concentration ≥45 kU/L [21] and positive specific IgE ≥0.35 kU/L (ImmunoCAP; Uppsala, Sweden).

### Statistical analysis

The chi-square test and Fisher's exact test were used to compare the distribution of each variable (clinical, environmental and environmental) between the control and the AR group. A logistic regression analysis [22] was used to examine the relationships between the binary outcome of interest (presence of AR) and multiple risk factors. Univariate and multivariate models, with and without interaction terms, were constructed to better understand the presence of AR. Spearman's correlation coefficient was computed to examine the association between variables. Risk factors associated with AR in the univariate analysis (p<0.2) were included in the multivariate analysis. The multivariate models were selected using the backward stepwise procedure.

A random forest analysis [23] was then applied to classify AR. This ensemble method uses a number of classification trees [24] to improve the classification compared to a single tree. In addition to the good predictive performance, random forests estimate the relevance (discriminating power) of each variable using importance measure (permutation-based mean decrease in accuracy). This non-linear approach provides an alternative to the logistic regression by providing another variables selection. Statistical analysis was performed with R version 2.12.0 (http://www.r-project.org). The R package ‘glm’, ‘Rpart’ and ‘randomForest’ were used to perform the analysis.

## Results

Forty-one of the 227 children seen in the TAP between January and November 2011 (18.1%) had AR. Among the remaining 186 asthmatic children without AR, 88 were randomized and enrolled in the control group. The two groups were similar regarding age (17 [6]-[30] versus 16 [6]–[29] months in the AR and control group respectively) and gender (54% boys in the AR group vs. 53% in the control group).

### Risk factors for AR: descriptive analysis (Table 1, Table 2)

**Table 1:** Characteristic features of the entire population, the control group and the AR group.

	Entire population (n = 129)	AR group (n = 41)	Control group (n = 88)	p-value*
Eczema	43 (33.0)	18 (43.9)	25 (28.4)	0.124
Food allergy	11 (8.5)	7 (17.1)	4 (4.5)	0.042
Moderate to severe Asthma	66 (51.2)	26 (63.4)	40 (45.4)	0.080
Parental history of Asthma	3 (2.3)	0 (0)	3 (3.4)	0.770
Parental history of AR	12 (9.3)	6 (14.6%)	6 (6.8)	0.119
Eosinophilia (≥470/mm^3^)	30 (23.3)	10 (24.4)	20 (22.7)	0.896
Elevated total IgE (≥45 kU/L)	38 (29.5)	12 (29.3)	26 (29.5)	0.885
Phadiatop infant	36 (27.9)	20 (22.7)	16 (39.0)	0.071
Polysensitization	20 (15.5)	11 (26.8)	9 (10.2)	0.030
Sensitizations to food and aeroallergens (≥0.35 kU/L)				
Cow Milk	24 (18,6)	11 (26.8)	13 (14.8)	0.163
Egg	22 (17.1)	11 (26.8)	11 (12.5)	0.078
Peanut	14 (10.9)	10 (24.4)	4 (4.5)	0.002
House dust mite	10 (7.8)	4 (9.8)	6 (6.8)	0.820
Cat	6 (4.7)	3 (7.3)	3 (3.4)	0.381
Dog	1 (0.8)	1 (2.4)	1 (1.1)	0.999
Grass pollen	2 (1.6)	1 (2.4)	1 (1.1)	0.536
Birch pollen	1 (0.8)	1 (2.4)	0 (0)	0.318
Alternaria	2 (1.6)	2 (4.9)	0 (0)	0.099
Any aeroallergen	1 (8.5)	5 (12.2)	6 (6.8)	0.497

All the values are expressed in absolute numbers and percentage in brackets. *Chi-square test when conditions were respected or Fisher's exact tests otherwise. Boldface values indicate statistical significance. Food allergy was defined by clinical symptoms of allergy after consumption of a food allergen and a positivity of specific IgE for the same allergen. Severity of asthma was determined according to the National Asthma Education and Prevention Program [20]. Polysensitization is defined as two or more specific allergen sensitizations.Any aeroallergen is defined as at least one aeroallergen sensitization.

**Table 2:** Environmental characteristics of the entire population, the control group and the AR group.

	Entire population (n = 129)	AR group (n = 41)	Control group (n = 88)	p-value*
Breastfeeding	69 (53.5)	23 (56.1)	46 (52.3)	0.829
Overcrowding	12 (9.3)	2 (4.9)	10 (11.4)	0.336
Mold exposure	33 (25.6)	18 (43.0)	15 (17.0)	0.002
Cockroaches	9 (7.0)	3 (7.3)	6 (6.8)	0.999
Passive smoking	35 (27.1)	13 (31.7)	22 (25)	0.558

All the values are expressed in absolute numbers and percentage in brackets *Chi-square test when conditions were respected or Fisher's exact test otherwise. Boldface values indicate statistical significance. Maternal breast-feeding beyond 3 months, Passive smoking (defined by smokers at home smoking more than 5 cigarettes a day). Overcrowding (less than 9 m^2^ for each person living at home). Mold exposure (visible mold and moldy smell) and cockroaches (visible cockroaches at home).

The AR group appeared to have –more features of allergy: food allergy (p = 0.042), allergic polysensitization (p = 0.030), especially due to peanut sensitization (p = 0.002) and greater exposure to mold (p = 0.030). Manifestations of food allergy were cutaneous (rash or facial edema) for seven of them and digestive (nausea or vomiting) for three. One child had both types of symptoms.

### Risk factors for AR: univariate logistic regression analysis (Table 3)

**Table 3:** Univariate logistic regression in the entire population (n = 129).

	Odds ratio	95% CI	p-value*
Eczema	1.97	0.91–4.27	0.084
Food allergy	4.32	1.19**–**15.73	0.026
Moderate to severe Asthma	1.40	0.94–2.10	0.098
Parental history of AR	1.42	1.01**–**2.02	0.046
Mold exposure	3.81	1.66**–**8.74	0.002
Phadiatop infant	2.27	1.01**–**5.07	0.046
Polysensitization	3.22	1.21**–**8.54	0.019
Cow milk sensitization	2.11	0.85–5.24	0.105
Egg sensitization	2.56	1.01**–**6.55	0.048
Peanut sensitization	6.77	1.98**–**23.19	0.002

*Wald test. Only p-value<0.2 are reported. Boldface values indicate statistical significance.

Food allergy was defined by clinical symptoms of allergy after consumption of a food allergen and a positivity of specific IgE for the same allergen. Moderate to severe asthma was determined according to the National Asthma Education and Prevention Program [20]. Mold exposure: visible mold and moldy smell. Allergen sensitization: specific IgE ≥0.35 kU/L. Polysensitization: defined as two or more specific allergen sensitizations.

In univariate logistic regression analysis, AR was found to be mainly associated with food allergy (OR = 4.31; p = 0.026); peanut (OR = 6.75; p = 0.002) and then with one environmental factor: mold exposure (OR  = 3.81; p<0.01). Other risk factors were found to be: biological biomarkers identified by Phadiatop infant (OR  = 2.27; p = 0.046), more precisely polysensitization (OR = 3.22; p = 0.019); and egg sensitization (OR = 2.56; p = 0.048); and finally a parental history of AR (OR = 1.42; p = 0.046).

### Risk factors for AR: multivariate logistic regression analysis (Table 4)

In the multivariate analysis, model selection using an automatic stepwise procedure led to a model without interaction for the following variables: food allergy (OR_a_ = 3.96; p = 0.081), peanut sensitization (OR_a_ = 3.40; p = 0.075), mold exposure (OR_a_ = 3.20; p = 0.013), familial history of AR (OR_a_ = 1.45; p = 0.052) and moderate to severe asthma (OR_a_ = 1.24; p = 0.124) (model 1). A correlation study highlighted the association between food allergy and peanut sensitization (χ^2^ = 11.23; p<0.001). Such a correlation should be avoided as the two variables share common information. We thus constructed a model without peanut sensitization (Model 2) and a model without food allergy (Model 3) (Table 4). The OR associated with peanut sensitization or food allergy were found to be higher in these models. After adjustment, peanut sensitization, food allergy, parental history of AR and the presence of mold were found to increase the risk of AR.

**Table 4:** Multivariate logistic regression in the entire population (n = 129).

	Model 1 OR (95% CI) [p-value*]	Model 2 OR (95% CI) [p-value*]	Model 3 OR (95% CI) [p-value*]
Food allergy	3.96 (0.84-18.57) [0.081]	5.96 (1.43**–**24.84) [0.014]	
Peanut sensitization	3.40 (0.86**–**13.07) [0.075]		5.02 (1.38**–**18.25) [0.014]
Mold exposure	3.20 (1.28**–**7.97) [0.013]	3.84 (1.59**–**9.28) [0.003]	2.86 (1.18**–**6.94) [0.020]
Parental history of AR	1.45 (0.99–2.12) [0.052]	1.46 (1.01**–**2.13) [0.043]	1.41 (0.97–2.05) [0.073]
Moderate to severe asthma	1.40 (0.91–2.17) [0.124]	1.41 (0.92–2.16) [0.117]	1.39 (0.90–2.14) [0.136]

*Wald test. Risk factors associated with AR in the univariate analysis (p<0.2) are included in the multivariate analysis. Automatic backward stepwise procedure was used for model selection (Model 1). Due to the correlation between food allergy and peanut sensitization, one of these variables is removed from the selected model (Model 2 and Model 3). Boldface values indicate statistical significance.

Food allergy was defined by clinical symptoms of allergy after consumption of a food allergen and a positivity of specific IgE for the same allergen. Moderate to severe asthma was determined according to the National Asthma Education and Prevention Program [20]. Allergen sensitization: specific IgE ≥0.35 kU/L. Mold exposure: visible mold and moldy smell. Cockroaches: visible cockroaches at home.

### Risk factors for AR: random forest analysis

**Figure 1:**
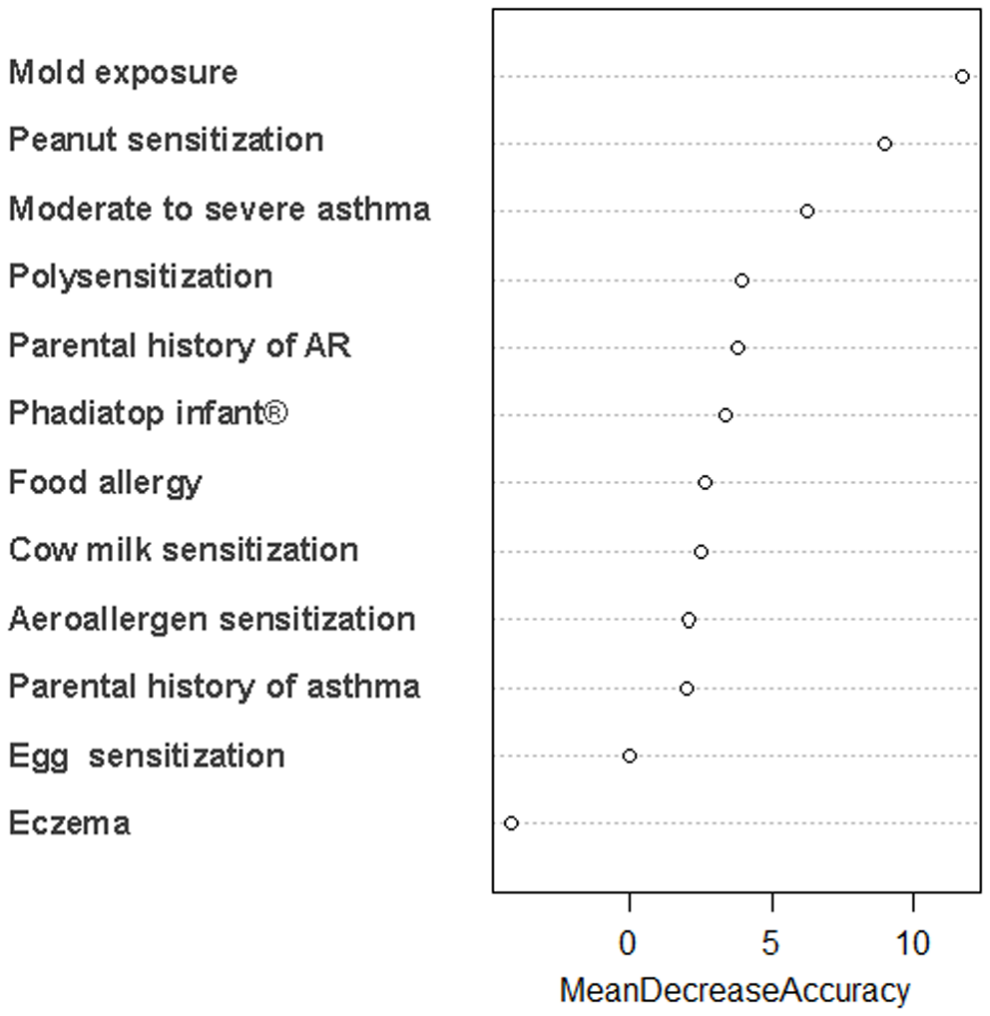
Importance measure (permutation-based mean decrease in accuracy) provided by the random forest analysis. Highest values of the importance measure are associated with a higher discriminating power.

The importance measure (permutation measure) obtained from the random forest analysis (Fig. 1) can be interpreted as a measure of discriminating power. These measures reinforced the previous results of peanut sensitization, mold exposure, moderate to severe asthma, parental history of AR, polysensitization and food allergy being determinants of AR. The mold exposure and peanut sensitization were associated with the highest values and seems to be the most important variables to explain AR.

## Discussion

The main result of our study is that independent variables for AR in young asthmatic children are mainly peanut sensitization (linked to food allergy) and a moldy environment. Taking together all the results, in a less constant manner, an association was found between AR and moderate to severe asthma but also a parental history of AR and polysensitization.

### Sensitization to peanut results in a fivefold increase in the risk of AR

In school age children, co-morbidities of allergic diseases such as AR and asthma occur more frequently in sensitized children (especially related to aeroallergen) [25], [26]. The fact that biological evidence of atopy should lead to a suspicion of AR in infants has already been shown by Herr *et al.* [13] but without identifying a specific allergic sensitization.

In our study, peanut sensitization appeared to be an important determinant of AR in preschool children. Similarly, Kulig *et al.* highlighted the fact that sensitization to a food allergen is a risk factor for further sensitization to an aeroallergen even without clinical food allergy [27]. Moreover, a particular link between peanut sensitization and the severity of allergic disease (such as eczema) has recently been described in the LEAP (Learning Early About Peanut Allergy) study [28]. Finally, it has also recently been described that a certain amount of peanut present in home air could stimulate basophils under IgE immune response [29]. These results, along with ours, point up the possibility that inhaled peanut could be a specific trigger of AR.

Moreover, we showed a significant link between food allergy and AR within the same toddler period. The relatively low importance of food allergy in random forest analysis was explained by the correlation with peanut sensitization [30]. This association between food allergy and allergic respiratory disease could simply reflect a particular severe allergic phenotype in young children with asthma, previously described by our team [17].

### Mold exposure an environmental determinants of AR

Our findings identified indoor dampness and the resulting molds as important risk factors for AR in young children with asthma. This risk has also been pointed out by the ISAAC study in the 6 to 7 year-old age group [31]. A Finish cohort of children followed from age 1 to 7 years also linked mold to the *de novo* development of AR (OR = 1.96; CI 95%: 1.29–2.99) and showed the increased risk to be dose-dependent [32]. There are certainly multiple mechanisms involved. Molds enhance direct sensitization with specific IgE production. However, we believe that the detrimental exposure is more due to irritation than to allergic sensitization towards mold, especially in young children. In fact, it is well known that mold exposure promotes bacterial growth and the release of volatile organic compounds which enhance non-specific inflammation especially in severe asthmatic children [33], [34]. The increase of epithelial permeability could thus lead to sensitization to other aeroallergens [35].

### Influence of a parental history of AR

Our study shows a link between the development of AR in early childhood and a parental history of AR. This result is in line with previous findings. The follow-up of the PARIS birth cohort estimated that a double parental history is associated with a twofold increased risk of the infant developing AR. In a study of children in Singapore, the risk of AR was 4.5 times higher (CI 95%: 3.3–6.1) when both parents had AR [36]. This transmission of a precise phenotype has also been observed for atopic dermatitis and asthma. In the English ALSPAC cohort, a history of atopic dermatitis in both parents was associated with an OR of 2.72 (CI 95%: 2.09–3.53) in children with atopic dermatitis [37]. Furthermore, the PARIS birth cohort showed that infants having an atopic asthma phenotype were more likely to have asthmatic parents [38]. The awareness of this phenotypic link could sensitize health professionals to the diagnosis of AR in asthmatic infants and improve their treatment and follow-up. In the same way, we recently found an association between mold exposure and AR present in a particularly severe allergic phenotype in young children with asthma [17].

### Severe asthma and its link with AR

A link between AR and moderate to severe asthma as independent variables was found by random forest analysis with a borderline association in the three multivariate logistic regression models. It has been well reported in the literature that AR worsens the asthma prognosis [38], while intranasal treatment improves asthma outcomes [39]. Moreover, bronchial hyper-responsiveness is frequent in children with rhinitis especially in those with AR [40]. Finally, AR is also more likely to persist during childhood than non AR [41].

### Polysensitization, another risk factor of AR

Simpson *et al.* [42] showed that atopic sensitization is not a dichotomous trait in relation to allergic respiratory disease (especially asthma). They demonstrated that a particular phenotype associated with multiple early allergic sensitizations predicts not only the presence, but also the persistence and severity of childhood asthma.

On a clinical level, Schröder *et al.* [43] confirm this point of view, with a significant association between food allergy and risk of asthma in children from a family-based food allergy cohort. Moreover, in accordance with our study, they demonstrated that the association was stronger in children with multiple food allergies.

### Strengths and limitations of the study

The main strength of this study lies in the use of precisely defined clinical parameters and biological markers. AR was assessed by medical doctors, supported by the SFAR criteria, which were more stringent than those used in previous studies [13]. Moreover, we had access (1) to a relatively large group of asthmatic toddlers with AR; (2) who shared close characteristics to toddlers with AR described in general population by Herr *et al.* [13]. Nevertheless, a limitation of our study is that we did not perform food challenges for diagnosing food allergies. Another limitation is that our questionnaire could have been more precise regarding environmental factors by using objective measurements. This fact may explain why our results did not show any link between AR and passive smoking, contrary to other authors [7], [44].

## Conclusions

Our study reveals that in young children with asthma, AR could be a signal symptom of a severe allergic phenotype with a particular association with peanut sensitization. This data stresses the importance of thoroughly investigating AR so as not to miss potentially diagnosing a particular severe asthmatic phenotype. Finally our results raise the hypothesis that peanut could be considered as an inhaled allergen.
