# Correction: Determinants of Allergic Rhinitis in Young Children with Asthma

**DOI:** 10.1371/journal.pone.0107414

**Published:** 2014-09-02

**Authors:** The PLOS ONE Staff

The fifth author’s name is spelled incorrectly. The correct name is: Flore Amat. The correct citation is: Moussu L, Saint-Pierre P, Panayotopoulos V, Couderc R, Amat F, et al. (2014) Determinants of Allergic Rhinitis in Young Children with Asthma. PLoS ONE 9(5): e97236. doi:10.1371/journal.pone.0097236
